# Recent expansion of marine protected areas matches with home range of grey reef sharks

**DOI:** 10.1038/s41598-021-93426-y

**Published:** 2021-07-09

**Authors:** Lucas Bonnin, David Mouillot, Germain Boussarie, William D. Robbins, Jeremy J. Kiszka, Laurent Dagorn, Laurent Vigliola

**Affiliations:** 1 Laboratoire d’Excellence Labex Corail, UMR IRD-UR-CNRS ENTROPIE, Centre de Nouméa, IRD (Institut de Recherche pour le Développement), BP A5, 98800 Nouméa Cedex, New Caledonia France; 2 MARBEC, CNRS, Univ Montpellier, Montpellier, Ifremer, IRD France; 3 Australian Research Council Centre of Excellence for Coral Reef Studies, James Cook University, Townsville, QLD 4811 Australia; 4 Wildlife Marine, Perth, WA 6020 Australia; 5 Department of Environment and Agriculture, Curtin University, Perth, WA 6102 Australia; 6 School of Life Sciences, University of Technology Sydney, Sydney, NSW 2007 Australia; 7 Department of Biodiversity, Conservation and Attractions, Marine Science Program, Biodiversity and Conservation Science, Kensington, WA 6151 Australia; 8 Institut of Environment, Department of Biological Sciences, Florida International University, 3000 NE 151st Street, North Miami, FL 33181 USA

**Keywords:** Behavioural ecology, Conservation biology

## Abstract

Dramatic declines in reef shark populations have been documented worldwide in response to human activities. Marine Protected Areas (MPAs) offer a useful mechanism to protect these species and their roles in coral reef ecosystems. The effectiveness of MPAs notably relies on compliance together with sufficient size to encompass animal home range. Here, we measured home range of 147 grey reef sharks, *Carcharhinus amblyrhynchos*, using acoustic telemetry in New Caledonia. The distribution of home range was then compared to local MPA sizes. We report a home range of 12 km^2^ of reef for the species with strong differences between adult males (21 km^2^), adult females (4.4 km^2^) and juveniles (6.2 km^2^ for males, 2.7 km^2^ for females). Whereas local historic MPA size seemed adequate to protect reef shark home range in general, these were clearly too small when considering adult males only, which is consistent with the reported failure of MPAs to protect sharks in New Caledonia. Fortunately, the recent implementation of several orders of magnitude larger MPAs in New Caledonia and abroad show that recent Indo-Pacific MPAs are now sufficiently large to protect the home ranges of this species, including males, across its geographical range. However, protection efforts are concentrated in a few regions and cannot provide adequate protection at a global scale.

## Introduction

Reef sharks are among the largest resident predators on coral reefs, playing a variety of ecological roles that could potentially be important for reef communities^1–3^. Due to their conservative demography, reef sharks are particularly vulnerable to anthropogenic mortalities from fisheries, particularly overfishing and bycatch^4–6^. Dramatic declines of populations have been documented worldwide^6–11^, raising concern about the potential ecological impacts of the extirpation of these predators in coral reef and other ecosystems^2,6,12^.

Excluding fishing activities through the implementation of Marine Protected Areas (MPAs) has often been proposed as the main solution to prevent the collapse of reef shark populations^13–16^. However, the ability of MPAs, including no-take zones, to protect mobile top predators such as reef sharks is increasingly being questioned^17,18^. For instance, no-take MPAs have been reported to have almost no effect on reef shark abundance on the Australian Great Barrier Reef (GBR)^8^. Similarly, in the Chagos archipelago, one of the largest marine protected areas in the world, current abundances of reef sharks have been shown to be low compared to estimated baseline levels^11^. In New Caledonia (South-Western Pacific), even an established (40 years), relatively large (170 km^2^) and highly-restrictive (no-entry) MPA failed to protect or restore baseline levels of reef shark abundance^9^ and behaviour^19^.

The incidence of illegal fishing inside MPAs is believed to be partly responsible for the lack of effectiveness in protecting reef sharks^8,11,13^. These species are indeed highly vulnerable to even low levels of anthropogenic mortality due to their life history traits^7,20^. In addition, fishing efforts tend to increase in the vicinity of MPAs, thereby increasing mortality in the proximate outside^21,22^. As such, besides the need for a strict enforcement of regulations within their boundaries, MPAs should be sufficiently large to limit the spread of individuals exiting to surrounding areas open to fishing. This can be an issue for mobile species such as sharks.

The inability of MPAs to encompass individuals’ movements has been identified as contributing to their failure to protect mobile species^18,23,24^. Through a meta-analysis of 87 MPAs around the world, size and isolation by deep water or sand have been highlighted as key factors in MPAs’ efficiency to protect predator species^25^, with only a small subset of these MPAs qualified as large (> 100 km^2^). For coastal sharks, MPAs > 20,000 km^2^ have been identified as the most efficient^6^. Recognizing that the effectiveness of an MPA to protect mobile species partly relies on its ability to encompass the home range of individuals, i.e. the area where animals spend most of their time^26–31^, a critical step in assessing the effectiveness of current MPAs for mobile reef shark species should thus consist in comparing individuals’ home ranges with the size of protected areas.

The grey reef shark, *Carcharhinus amblyrhynchos*, is one of the most common reef shark species in the Indo-Pacific^2,8,9,11,32–34^. The estimated home range for this species^35,36^, along with its high level of site residency^37–39^ and long-term fidelity^40–42^, suggest that MPAs > 100 km^2^ would be appropriate for its protection. However, in New Caledonia, such MPAs were assessed as failing to protect this species (Juhel 2017, 2019).

Since illegal fishing pressure is believed to be low in New Caledonia^9,19^, this inconsistency raises questions about the current assessments of grey reef shark home range. Indeed, current knowledge of home range and movement abilities of this species is often based on relatively small sample sizes, typically less than 40 individuals^38,39^, and female-skewed sampling^39–41^. Subsequent estimations of home ranges could then have overlooked individual variations with a loss of pertinent information for the assessment of MPA ability to protect all components of a shark population.

In order to overcome these limitations, 147 adult and juvenile grey reef sharks of both sexes were tracked with acoustic telemetry for over three years within an array of 70 acoustic receivers across the New Caledonian archipelago, where 25 protected areas of various sizes (3–30,000 km^2^), ages (established 1970–2018) and restrictions (no-take and no-entry) exist. The distributions of shark home range was calculated and compared to the size of MPAs in New Caledonia and in the Indo-Pacific to assess the extent to which MPAs encompass the majority of grey reef shark home range, and thus offer adequate protection for this species.

## Material and methods

### Study area

**Figure 1:**
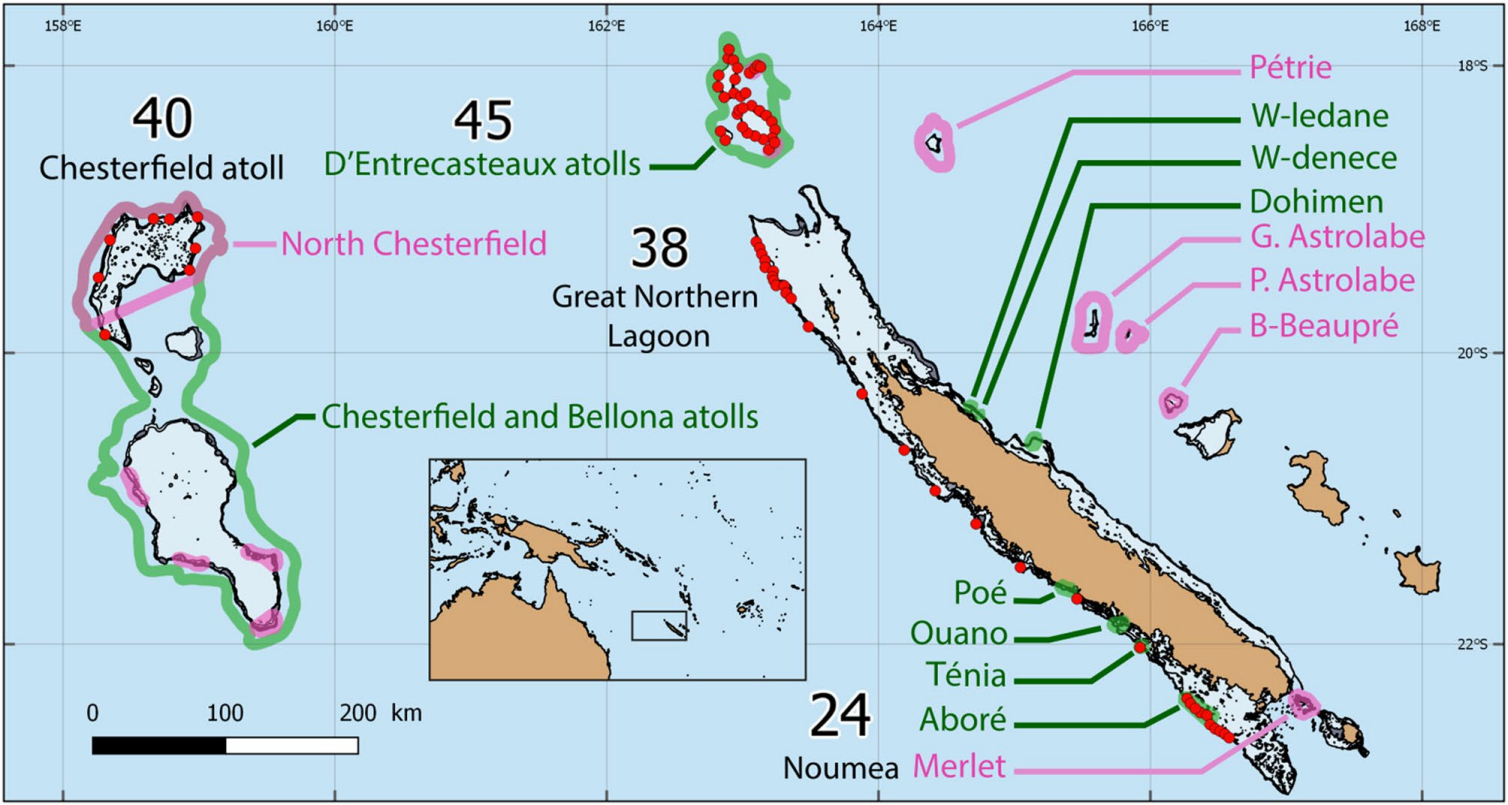
Acoustic array, marine protected areas and shark sampling in the New Caledonian archipelago, South-Western Pacific. Red circles indicate acoustic receivers. The number of sharks sampled in the four study regions are displayed. Green contours represent no-take MPAs and pink contours represent no-entry MPAs. Map generated with QGIS Version 3.2 (https://www.qgis.org/) and shapefile data from^43^.

New Caledonia is an archipelago consisting of isolated islands, atolls and reefs, with a 400 km × 60 km mainland, surrounded by a continuous barrier reef^43^. The archipelago notably includes the remote D’Entrecasteaux atoll group, separated from the northern part of the mainland lagoon by a 35 km wide and 500 m deep channel, and Chesterfield and Bellona atolls, located in the heart of the Coral Sea, 400 km offshore from the New Caledonia mainland, midway to Australia Great Barrier Reef. Twenty-five MPAs are currently established in New Caledonian waters (Table S1), with restriction levels ranging from the prohibition of all extractive activities such as fishing (no-take MPAs) to the prohibition of all human activities including the entrance of ships (no-entry MPAs). Among these, only 14 encompass the outer slopes of barrier reefs, the preferred habitat of grey reef sharks (Fig. 1, Table S1). Although grey reef sharks may be present in all habitats of a coral reef ecosystem, their movements and abundances clearly indicate that the outer slope is by far their preferred habitat^9,41,44^. These 14 MPAs include an ancestral no-entry customary sanctuary, the Beautemps-Beaupré atoll encompassing 160 km^2^, seven no-take MPAs implemented between 1993 and 2009 and ranging between 3 and 150 km^2^ in size and the no-entry Merlet reserve implemented in 1970 and encompassing 170 km^2^. They also include five newly created off-shore reserves (2018): three no-entry MPAs at Petrie atoll, Petit Astrolabe reef and Grand Astrolabe reef, respectively encompassing 600, 200 and 725 km^2^, and two no-take MPAs at the atolls of Entrecasteaux and Chesterfield & Bellona, encompassing respectively 3500 and 27,150 km^2^, and comprising several no-entry zones up to 6, 600 km^2^ (North-Chesterfield, Table S1).

### Shark tagging, acoustic array and raw data filtering

A total of 147 grey reef sharks were internally fitted with V16 acoustic transmitters (68 mm × 16 mm; frequency: 69 kHz; high power output; transmission delay times: random between 30 and 90 s). Seventy VR2W acoustic receivers (VEMCO Ltd., Halifax, Canada) were deployed from July 2015 to December 2018 across four regions of the New Caledonian archipelago (D’Entrecasteaux, Chesterfield, Great Northern Lagoon [GNL], Noumea; Fig. 1). Receivers were deployed along the outer slope of the barrier reef, where sharks were caught and tagged. Sharks were caught with floating drumlines. Total length, sex, and maturity were determined based on the extension and calcification for males, and extrapolated from total length according to^20^ for females. Further information on shark tagging and acoustic receiver deployment procedures are provided in Appendix S2. Raw acoustic data was filtered to remove potential false detections using the *FDA Analyzer Tool* from the *VUE* software (VEMCO Ltd., Halifax, Canada). All methods, including shark capture, handling, tagging and release, were carried out in accordance with relevant guidelines and regulations in New Caledonia. All experimental protocols were approved and authorised by the Government of New Caledonia (permit 2015-1351/GNC), the Southern Province of New Caledonia (permit 479-2016/ARR/DENV and 2093-2016/ARR/DENV) and the Northern Province of New Caledonia (permit 60912-1508-2015/JJC).

### Estimating shark home range

Individual home range can be estimated through the calculation of Utilization Distributions (UD). Kernel-based density methods are usually used to calculate UD^36,45–47^. Yet, the linear nature of our array system along the grey reef shark main habitat, and the resolution associated with the spacing between acoustic receivers did not allow kernel-based methods (Fig. 1). Instead, individual UDs were calculated as one-dimensional convex hulls defined by the portions of outer reef slope delimited by the locations of visited receivers. The 95^th^ and 100^th^ percentile of daily positions were used to determine UD boundaries. This approach is similar to the metric used to estimate crocodile home range along other relatively linear systems such as rivers^48^. In order to identify receivers associated to the 95^th^ percentile boundary, the monitoring period was split into daily bins and occurrence within each bin was assigned to a receiver if the animal was detected at this receiver that day. Receivers were then ranked according to the number of daily occurrences and a subset was selected according to a 95% threshold. UD_95_ and UD_100_ were calculated as the surface of outer reef slope encompassed by the reef portion delimited respectively by the 95th percentile subset of receivers and all visited receivers (Fig. S1). The outer reef slope habitat was identified as fore reef, reef pass and subtidal reef flat within barrier reef complexes, using coral reef habitat data provided by^43^. When individuals travelled between separated reefs, they were conservatively considered to have travelled the shortest straight-line distance between the two reefs.

### Sex, ontogenetic and seasonal variability

Two-way PERMANOVAs were performed to test if UD_95_ and UD_100_ values varied between sex and maturity stage (juvenile or adult). The *aovp* function from the *lmPerm R* package was used, considering the Anscombe criterion to determine the number of permutations. Pairwise permutation Student tests (n = 999 permutations) were performed to compare the UD_95_ and UD_100_ values of adult males, adult females, juvenile males and juvenile females. UD values were log-transformed (*y* = log_10_(1 + *x*)) before analyses.

Seasonal variations in shark home range were investigated by identifying reef portions used during the mating season (July–September^34^), outside the mating season (October–June), or during both seasons. Then, the percentage of UD_100_ exclusively used during the mating season was computed and compared among sex and maturity stage by PERMANOVA. UD_95_ and UD_100_ values were then computed for each season and compared among sex and maturity stage by PERMANOVA.

Cumulative UD_95_ and UD_100_ curves were also built for the different sexes and stages using a random sampling procedure without replacement and a bootstrap procedure (200 runs) to determine 95% confidence intervals.

### Assessing MPA’s ability to cover shark home range

Distributions of UD values were used to model the ability of an MPA covering a surface *S* of outer reef slope to cover shark home range. We first discretized *S* in *n* intervals of size *dS* = 1 km^2^. The probability *P*_*ij*_ that the UD_*j*_ of a shark *j* centred in interval *i* would be fully covered by protection was set to one when the UD_*j*_ of shark *j* centred in *i* was fully covered by the MPA, and set to zero otherwise. For this interval *i*, a probability *P*_*i*_ was calculated as the average of *{P*_*ij*_*}* for all shark *j* from the distribution. Then, the probabilities *{P*_*i*_*}*_*n*_ for all interval *i* encompassed by the *S*-large protected area were averaged to estimate the probability Π that the UD of any shark, located at any place in the protected area, would be fully covered (Fig. S2). This probability was modelled for UD_95_ and UD_100_ separately, and considering the distribution of UD values over all sampled sharks and for adult males only.

In order to test if existing MPAs encompassed grey reef shark home ranges, we calculated surfaces of reef slopes covered by existing MPAs in New Caledonia and across sites in the Indo-Pacific, using the World Database of Protected Areas^49^. As reef typology was not available to identify the outer reef slope habitat in the entire Indo-Pacific, plain reef habitat from the UNEP-WCMC was used instead^50^. As non-reef slope habitat was included in these estimates, this gave a conservative estimate of the extent of critical MPA-habitat. Individual MPA polygons were selected from the WDPA if they encompassed reef habitat, had no extractive activities (IUCN categories I and II), had their centroid comprised between latitudes 26°S and 26°N and between longitudes 25°E and 150°W (grey reef shark geographical range) and if their year of creation was informed. Inclusion of MPAs from New Caledonia resulted in a non-exhaustive dataset of 622 MPAs.

Among the 147 grey reef sharks fitted with acoustic transmitters (Fig. 1), 29 showed no detection after a two-week post-capture period and were subsequently excluded from analyses. Three other individuals were also excluded because the receiver they were tagged at was lost, preventing appropriate home range calculation. Analyses were therefore performed on 118 individuals, including 53 adult males, 19 adult females, 19 juvenile males and 27 juvenile females.

Using the same acoustic telemetry dataset, six adult males have been observed to undergo long-range migrations in New-Caledonia, up to 300 km from their tagging site^34^. These individuals were included in the home-range analyses presented hereafter, but analyses were also performed without these individuals to ensure results were not biased by long-range migrators.

## Results

### Home range estimates and variability

PERMANOVA revealed a significant effect of both sex and maturity stage on home range for both UD_95_ and UD_100_ metrics (Table 1). Overall, sharks showed a mean UD_95_ of 6 km^2^ (2.2–9.2 km^2^ 95% bootstrapped CI) and a mean UD_100_ of 12 km^2^ (7.3–15.7 km^2^ CI) of reef. Adult males had significantly and substantially larger home range (UD_95_ = 13 km^2^ [4.9–19.2 km^2^ CI], UD_100_ = 21 km^2^ [12–18 km^2^ CI]) than adult females (UD_95_ = 0.72 [0.06–1.2 km^2^ CI], UD_100_ = 4.4 km^2^ [2.6–6.0 km^2^ CI]), juvenile males (UD_95_ = 1.0 km^2^ [− 0.15–1.9 km^2^ CI], UD_100_ = 6.2 km^2^ [− 1.4–10.8 km^2^ CI]) and juvenile females (UD_95_ = 0 km^2^ [0–0 km^2^ CI], UD_100_ = 2.7 km^2^ [− 0.5–4.8 km^2^ CI], Fig. S3).

**Table 1:** Explaining variability in shark home range by PERMANOVA tests.

Home range metric	Season		DF	Sum of squares	Mean square between	Iterations	P (perm.)
UD_95_	Year-long	Sex	1	8.752	8.752	5000	0.003**
		Maturity stage	1	7.363	7.363	4075	0.024*
		Sex:Mat. stage	1	1.890	1.890	539	0.158
		Residuals	114	137.672	1.208		
	Mating season (July–September)	Sex	1	10.622	10.622	5000	0.003**
		Maturity stage	1	11.487	11.487	5000	0.004**
		Sex:Mat. stage	1	7.510	7.510	5000	0.014*
		Residuals	114	156.303	1.371		
	October–June	Sex	1	2.115	2.115	1343	0.070
		Maturity stage	1	1.196	1.196	709	0.124
		Sex:Mat. stage	1	0.060	0.060	51	1.000
		Residuals	114	70.555	0.619		
UD_100_	Year-long	Sex	1	12.646	12.646	5000	0.001***
		Maturity stage	1	23.930	23.930	5000	0.000***
		Sex:Mat. stage	1	0.990	0.990	368	0.215
		Residuals	114	168.753	1.480		
	Mating season (July–September)	Sex	1	17.034	17.034	5000	0.000***
		Maturity stage	1	11.994	11.994	5000	0.003**
		Sex:Mat. stage	1	10.636	10.636	5000	0.007**
		Residuals	114	162.837	1.428		
	October–June	Sex	1	2.201	2.201	51	0.882
		Maturity stage	1	8.998	8.998	5000	0.003**
		Sex:Mat. stage	1	3.812	3.812	2334	0.041*
		Residuals	114	127.697	1.120		

Asterisks indicate *p*-value thresholds (***: < 0.001; **: < 0.01; *: < 0.05).

Two metrics of home range (UD_95_ and UD_100_) were compared between sex (male, female) and maturity stage (adult, juvenile) at all seasons, during the mating season, and outside the mating season.

Interestingly, adult males expanded their home range during the mating season between July and September, with a significant sex-maturity interaction on the proportion of outer reef slope exclusively used during this season (PERMANOVA, *p* = 0.0024). On average, adult males used 52% of their home range exclusively during the mating season, compared to only 9% for other groups (Fig. S4). Adult females and juveniles of both sexes did not show range expansion during the mating season, as 89% of them did not explore any new reef portions during this season.

**Figure 2:**
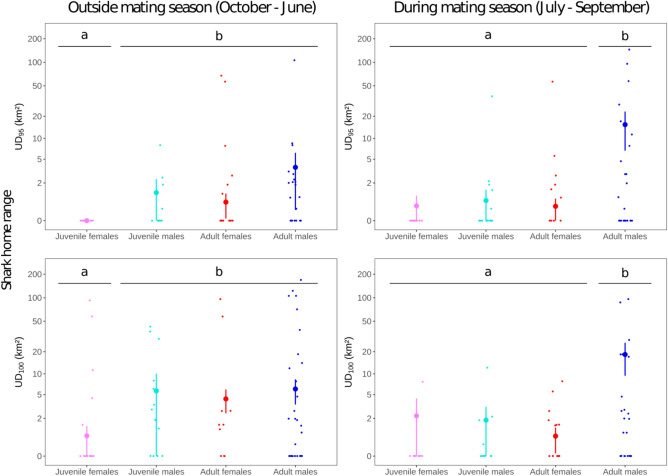
Comparison of home range for grey reef shark adults and juveniles during and outside mating season. UD_95_ and UD_100_ values represent the surface of outer reef slope habitat encompassed by the 95th and 100th percentile of daily positions. Large dots and bars indicate group means and their bootstrapped 95% confidence intervals. Significance of difference between group means were assessed with pairwise permutation Student tests and displayed with lower case letters. Graphics generated with R package ggplot2 (https://ggplot2.tidyverse.org).

Outside the mating season, home range did not significantly vary among sexes (UD_95_: *p* value = 0.07; UD_100_: *p* value = 0.882). However, maturity stage had a significant effect on UD_100_ during this season (*p* value = 0.003), with adults showing a significantly higher home range than juveniles (Table 1). During the mating season, both sex and maturity stages had significant effects on both home range metrics (UD_95_: sex *p* value = 0.003, maturity stage *p* value = 0.004; UD_100_: sex *p* value < 0.001, maturity stage *p* value = 0.003), with adult males showing significantly higher home ranges than adult females and juveniles of both sexes (Fig. 2).

Removing the six long-range adult male migrators from the analyses showed similar results (Table S2).

Cumulative curves of UD_95_ and UD_100_ values highlighted that 93% (95% bootstrapped-CI: 89–97%) and 76% (95% bootstrapped-CI: 69–83%) of sampled sharks had UD_95_ and UD_100_ values lower than 10 km^2^ of reef, respectively. When considering adult males only, respectively 83% (95% bootstrapped-CI: 74–94%) and barely half of individuals (54%; 95% bootstrapped-CI: 43–66%) had UD_95_ and UD_100_ values lower than 10 km^2^ of reef (Fig. S5).

### MPAs ability to cover sharks’ home range

**Figure 3:**
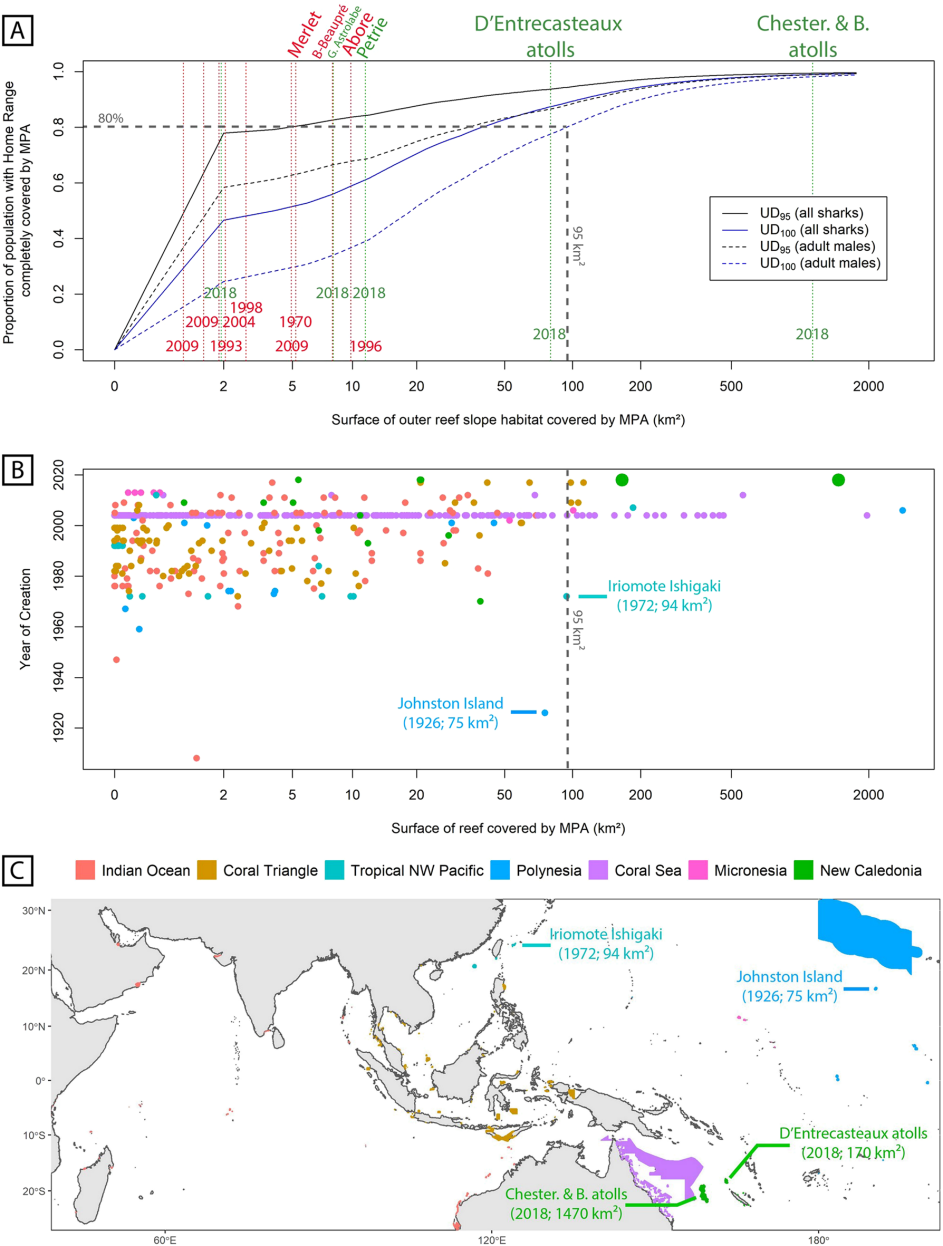
Marine Protected Areas (MPAs) ability to protect grey reef shark home range according to their size. (**A**) Distribution of home range values were used to model the ability of an MPA covering a given area of outer reef slope to cover sharks home range. This ability was modelled considering all sharks (plain lines) or adult males only (dotted lines). Black and blue lines present the ability of MPAs to cover sharks’ UD_95_ and UD_100_ respectively. New Caledonian MPA sizes are displayed with vertical dashed lines, in red for old MPAs (before 2010) and in green for recent ones. (**B**) Indo-Pacific MPAs from the World Database of Protected Areas (UNEP-WCMC, 2014) classified according to creation year and covered area of reef. (**C**) Location of Indo-Pacific MPAs. Graphics generated with R package ggplot2 (https://ggplot2.tidyverse.org) and rgdal (https://CRAN.R-project.org/package=rgdal).

Cumulative curves of UD values were then used to model the proportion of sharks whose home range could be fully covered by an MPA of a given size, considering a homogeneous distribution of individuals in the area (Fig. 3A). The model indicates that old MPAs of New Caledonia (created before 2010) were unable to cover the home range of grey reef sharks. For instance, the model shows that an MPA with the size of *Aboré* (a total area of 150 km^2^ but only covering 10 km^2^ of outer reef slope habitat) would be able to fully cover the UD_95_ for 84% of sharks, and the UD_100_ for 59% of sharks only (Table S3). These proportions dropped to 68% for UD_95_ and 37% for UD_100_ when considering adult males only. However, newly created MPAs of D’Entrecasteaux atolls and of Chesterfield and Bellona atolls, covering respectively 80 km^2^ and 1130 km^2^ of outer reef slope habitat (for a total area of 3500 km^2^ and 27,150 km^2^, respectively), would encompass the UD_100_ for 78% and 98% of adult males, respectively.

In the Indo-Pacific, such very large MPAs, able to protect the home range of adult male grey reef sharks, were rare and mostly created recently (Fig. 3B). Indeed, our model indicates that only MPAs covering more than 95 km^2^ of reef can achieve the ambitious management objective of protecting the home range (UD_100_) of 80% of adult males. Only 26 out of the 622 Indo-Pacific MPAs exceeded this threshold. These 26 MPAs were all created after 2004. Only two old MPAs came close to this size: the *Iriomote Ishigaki National Park* and the *Johnston Island National Wildlife Refuge*, respectively created in 1972 and 1926 by Japan and the United States and covering 94 and 75 km^2^ of reef (Fig. 3C). Among the 26 recent MPAs covering more than 95 km^2^ of reef, 17 were established in Australia, four in Indonesia, two in New Caledonia, one in Taiwan, one in the Marshall Islands and one in Hawaii. D’Entrecasteaux and Chesterfield & Bellona MPAs both cover more than 95 km^2^ of reef, respectively 170 and 1470 km^2^, and were thus added to the dataset comprising very large Indo-Pacific MPAs. Most of the 26 very large MPAs were established in the Coral Sea (n = 19) and the Coral Triangle (n = 4), followed by Micronesia (n = 1), Tropical North-West Pacific (n = 1) and Polynesia (n = 1) (Fig. 3C). These very large Indo-Pacific MPAs with over 95 km^2^ of protected reef range from 145 km^2^ to 1.4 millions of km^2^ total protected area.

## Discussion

### Main findings

The lack of efficiency of MPAs implemented in New Caledonia before 2010 to protect reef shark populations^9,19^ may be attributed to a large suite of factors, such as illegal fishing^51,52^ and human proximity-induced fitness alterations^19^. However, we focused here on the hypothesis that MPA ineffectiveness may also be due to their small size and their failure to protect reef shark home range.

Our results revealed that until very recently, the size of MPAs in New Caledonia were insufficient to protect grey reef shark home range, especially adult males. This may explain in part local MPAs’ failure to restore levels of abundances observed at remote reefs^9^. However, our results confirm the relevance of recent efforts of the Government of New Caledonia in implementing 1–2 orders of magnitude larger MPAs in the remote D’Entrecasteaux, Chesterfield, and Bellona atolls. Beyond protecting reef sharks, such very large MPAs should be able to protect the function played by large and mobile fish, predators especially, and thus key ecosystem properties and functional diversity^17,53^.

According to our model, very large MPAs enable coverage of the home range of nearly, if not all, local grey reef sharks. Moreover, the outputs of our model are likely conservative since its framework did not allow to consider habitat limitations. With more than a hundred individuals tagged, none was observed to cross oceanic channels greater than 35 km (Appendix S6, Fig. S6). This further supports our conclusion that recent and very large MPAs such as Chesterfield, Bellona and D’Entrecasteaux encompass the entire home range of grey reef sharks. It is also noteworthy that, for the same reason, a smaller isolated MPA such as Beautemps-Beaupré might be more efficient than suggested by our model. Matching reserve limits with habitat limits is indeed often proposed as a key feature to prevent spill-over of individuals outside protection^25,54^.

Our results also highlight the greater vulnerability of adult males, which are characterized by larger home ranges than females and juveniles. In non-monogamous species, males’ importance in population dynamics is often considered as lesser than females’, as females are likely to find a mate irrespective of male density^55,56^. Moreover, in species where mating include male harassment^57^, male density can be deleterious for population growth^56^. Yet, males’ lesser importance for population dynamics remains theoretical and has not been confirmed in this species, thus should not be used to draw males out of conservation efforts. Moreover, grey reef shark adult males provide an essential function in promoting genetic diversity, as they provide most gene dispersal in this species^58,59^. In this perspective, we think that conservation management deserves particular focus on this population segment.

### Home range estimation

Grey reef sharks were monitored in the outer reef slope habitat only, as it is their preferred habitat^41,60^. Outer reef slopes were thus considered as a linear system, and movements were interpreted as mostly occurring in this habitat. A similar approach was used by^38,42^. This species often uses the pelagic and lagoon habitat as well^61^, thus our results potentially underestimate the real magnitude of their space use. Nevertheless, our conclusions about MPA size should remain valid as MPAs were considered in their ability to cover sharks’ home range on their most critical habitat at the least.

Thirty-seven individuals were detected on one receiver only, highlighting a second source of home range underestimation with our methodology. Indeed, this methodology was limited by the resolution of the acoustic array. As the Utilization Distribution of individuals detected on one receiver was likely lower than receiver spacing in the array, these individuals were attributed null values of UD. Yet, this skew of small UD values to null UD values was of little consequences over following modelling of MPA’s ability to cover shark range, considering our modelling framework and the use of cumulative curves of UD values.

This modelling procedure was similar to the one used in^28^ but a homogeneous distribution of individuals in the theoretical MPA was considered here. Each discretized MPA interval was considered independently from the others, which implied that there was no consideration for potential consequences of an overlap of home ranges between neighbouring intervals. However, such overlapping would not impair our reasoning because the grey reef shark is a relatively social species that is not known to generally exclude conspecifics from its territory^62–64^.

Grey reef shark movement patterns are well described in the literature and numerous acoustic telemetry studies revealed a high level of long-term site-fidelity^40–42^ and residency^37–39^ in this species. Our results concur with these findings, as 37 out of the 118 tagged animals were detected on one receiver only, the one they were tagged at. Estimates of home range size are also well documented for this species^35,36^ but values from previous studies are difficult to relate to ours, partly due to differences in the UD estimators that were used, but mainly due to differences in receiver arrays configuration. When we report an average UD_100_ of about 12 km^2^ of reef over 118 individuals in three years of study, Speed et al.^35^ reported an average UD of about 20 km^2^ total area over 2 individuals in two years (minimum convex polygon, that consider all recorded positions) and Udyawer et al.^36^ reported an average UD of about 30 km^2^ total area over 27 individuals in three years (Brownian Bridge Kernel UD, a statistical method that infer probability of presence on locations where positions were not actually recorded). Both these studies monitored sharks on an array covering the lagoon habitat in addition to the outer reef slope, making comparisons difficult. Still, given that such arrays cover much larger areas of available habitat than ours, our estimates were expected to be several times lesser than the one reported in these studies. These higher values could be explained by the larger sampling size of our study, potentially enabling to better reflect inter-individual variations and thus unveiling a larger range of values. It could also indicate the preference for grey reef sharks to travel along corridors where our receivers were deployed. The incidence of adult male values in our average population estimate could also play a role in this, as neither of these previous studies accounted for variations of home range with sex and ontogeny.

### MPA minimum requirements

The modelling of MPAs’ ability to protect grey reef shark home range was based on two metrics: UD_95_ and UD_100_. The determination of MPAs’ size requirements is thus affected by the choice of the home range metric to consider, and this choice depends on whether it is essential to cover areas where animals spend most of their time or the entirety of visited areas. This decision relies on the level of risk that animals are exposed to outside MPAs. Assuming that MPAs protect from mortality, if risk is considered to be proportional to the time spent outside MPAs, protecting the UD_95_ may be adequate. However, in cases of greater risk level (e.g. high surrounding fishing pressure), any time spent outside MPAs may be lethal for an individual, and protecting animals’ UD_100_ may reveal necessary. Species’ need for protection may also be higher at particular times of their life cycle, and a change in space use during periods of vulnerability would not necessarily translate into greater UD_95_ if such periods are short enough. In our case, adult males showed range expansion during mating season, most likely linked to reproduction. Yet, this spatial scale is not well described by UD_95_ values, and considering UD_100_ would be more appropriate to protect areas required by adult males for reproduction. This consideration may also partly explain why previous knowledge about the grey reef shark home range, whose estimates were based on most frequented areas^35,36^, was inconsistent with MPAs’ inability to protect this species in New Caledonia^9,19^.

With activity space metrics based on dispersal distances, Dwyer et al.^31^ implemented a similar modelling procedure to provide recommendations on MPA size requirements to protect several species of reef sharks. They conclude that a 30 km-wide MPA would be theoretically able to fully protect only 50% of grey reef sharks. These conclusions concur with ours, as the Abore MPA, which roughly cover 35 km of continuous barrier reef, is expected to cover the UD_100_ of 59% of grey reef sharks in our system.

Irrespectively of the home range metric considered, our results highlighted the adequacy of the recent MPAs implemented in the remote D’Entrecasteaux atolls and Chesterfield & Bellona atolls to protect grey reef sharks. Protecting such remote areas, which constitute de facto refuges in the way that they are already almost free from human activities, may seem superfluous, and is often even considered to mislead conservation efforts by preventing resources being spent on areas under higher threats^65^. Yet, a meta-analysis of 1800 tropical reefs have concluded that effective conservation of marine predators could only be attained in remote areas, as even small rates of illegal extraction in protected areas close to human populations can be deleterious to such vulnerable species^13^. Moreover, remoteness is becoming increasingly less of an obstacle for industrial fishers^16,66–68^, raising concerns about the future exploitation of wilderness reefs such as D’Entrecasteaux and Chesterfield in a context of raising fish price^69^. This should be particularly emphasized for the case of sharks, given the high value of their fins on Asian markets^70^.

Protecting large areas may arguably be feasible only in remote areas. Conservation management in areas closer to human activities may thus be achieved through alternative measures. For example, Schofiel et al.^71^ proposed seasonal zoning in a marine park in the island of Zakynthos, Greece, based on seasonal space use of loggerhead turtles, *Caretta caretta*, around a critical breeding site. Our results provide reliable information to support the relevance of implementing seasonal protection measures, e.g. fishing gear restrictions or local MPA expansion. Such seasonal measures are all the more necessary when considering the case of New Caledonian migratory adult males^34^, capable of travelling several hundreds of km during the mating season. The protection of these few individuals, perhaps anecdotal when considering total population dynamics, may yet be essential to enable gene flow and metapopulation resilience.

### Global trend

The implementation of MPAs for the protection of marine ecosystems is usually based on either conservation or optimization of fishery yields^72^. The latter has spillover toward non-protected areas as the primary objective, and calls for the implementation of numerous closely-spaced MPAs^54,73^. Here however, protection is essentially considered in the perspective of conservation. The objective is to sustain populations in protected areas and thus to prevent spillover of individuals toward open areas where mortality risk is increased. Within a conservation framework, our results concur with those of^6^ and call for the implementation of much larger MPAs to protect coastal sharks globally. The grey reef shark was used here as a case study because of its abundance, but these findings should illustrate the importance of implementing large MPAs for the conservation of other coastal shark species, with less documented but similar movement abilities and whose conservation status is of even greater concern^74^.

Expanding our conclusions to the Indo-Pacific region revealed that very few MPAs were large enough to protect a mobile reef shark species such as the grey reef shark. Furthermore, such very large MPAs were all implemented recently, from the years 2000’s. This Indo-Pacific trend, notably impelled by the Convention on Biological Diversity that set a target of 10% of global marine areas to be protected before 2020^75^ brings new hope for marine ecosystem conservation, and especially for mobile species like reef sharks. Such hope has yet to be moderated as (i) large Indo-Pacific MPAs are concentrated in one region, the Coral Sea, and Australia is responsible for establishing a large majority of them, and (ii) the existence alone of such MPAs does not necessarily warranty conservation efficiency. Other factors, such as a lack of monitoring and enforcement, poor governance or lack of local support, can indeed compromise MPAs efficiency^18,76–78^.

## Supplementary Information

Supplementary Information.
