# Supplementary material for: Recent expansion of marine protected areas matches with home range of grey reef sharks
Source: Sci Rep. 2021 Jul 9;11:14221. doi: 10.1038/s41598-021-93426-y (PMC8270914; doi:10.1038/s41598-021-93426-y)
Supplement: Supplementary file 1 — Supplementary Information. [file 41598_2021_93426_MOESM1_ESM.pdf]

Supplementary Material

Recent expansion of marine protected areas matches with home range of grey reef sharks

Authors

Lucas Bonnin[1,2], David Mouillot[2,3], Germain Boussarie[1,2], William D. Robbins[4,5,6,7], Jeremy J. Kiszka[8], Laurent Dagorn[2] and Laurent Vigliola[1]

Affiliations

[1]IRD (Institut de Recherche pour le Développement), Laboratoire d’Excellence Labex Corail, UMR IRD-UR-CNRS ENTROPIE, Centre de Nouméa, BP A5, 98800 Nouméa Cedex, New Caledonia, France.

[2]MARBEC, Univ Montpellier, CNRS, Ifremer, IRD, Montpellier, France.

[3]Australian Research Council Centre of Excellence for Coral Reef Studies, James Cook University, Townsville, QLD 4811 Australia.

[4]Wildlife Marine, Perth, Western Australia 6020, Australia.

[5]Department of Environment & Agriculture, Curtin University, Perth, Western Australia 6102, Australia.

[6]School of Life Sciences, University of Technology Sydney, Sydney, New South Wales 2007, Australia.

[7]Marine Science Program, Biodiversity and Conservation Science. Department of Biodiversity, Conservation and Attractions, Kensington, WA 6151 Australia

[8]Institut of Environment, Department of Biological Sciences, Florida International University, 3000 NE 151st Street, North Miami, FL33181, USA.

Correspondance to lucasbonnin@hotmail.fr

Table S1. Summary information about the 25 marine protected areas (MPAs) currently established in New Caledonian waters. Only 14 MPAs encompassed *the outer slope of barrier reefs, the preferred habitat of grey reef sharks. The remaining eleven are displayed in red. No-entry MPAs prohibit all human activities including the entrance of ships while no-take MPA allow non-extractive activities.*

MPA	Protection status	Area (km²)	Creation year
Whanga-Iedane	No-take	8.7	2009
Whan-denece Pouarape	No-take	2.6	2009
Ouano	No-take	37	2004
Petit Astrolabe	No-entry	200	2018
Poe	No-take	32	1993
Tenia	No-take	14	1998
Dohimen	No-take	36	2009
Merlet	No-entry	170	1970
Beautemps-Beaupré	No-entry	160	ancestral
Grand Astrolabe	No-entry	730	2018
Abore	No-take	150	1996
Petrie	No-entry	600	2018
D'Entrecasteaux atolls	No-take	3500	2018
*North-Chesterfield	No-entry	6580	2018
Chesterfield and Bellona atolls	No-take	27150	2018
Kuendu	No-take	0.4	1998
Roche percée	No-take	1.4	1993
Prony (2 sites)	No-take	1.5	1993
Canard islet	No-take	1.9	1989
Ile verte	No-take	2.1	1993
Bailly islet	No-take	2.2	1989
Signal islet	No-take	2.5	1989
Pewhane	No-take	3.7	2009
Maitre islet	No-take	6.3	1984
Hienga	No-take	6.6	2010
Larégnère islet	No-take	6.7	1989

**: The North-Chesterfield no-entry reserve is part of the Chesterfield & Bellona atolls MPA.*

S2. Shark tagging and acoustic array deployment procedures.

Shark tagging

147 grey reef sharks were internally fitted with V16 acoustic coded transmitters (68 mm x 16 mm; frequency: 69 kHz; high power output; transmission delay times: random between 30 and 90 s; VEMCO Ltd., Halifax, Canada). Sharks were targeted along the outer slope of the barrier reef, where the receivers were deployed. Sharks were caught on a 16/0 barbless, non-stainless, non-offset circle hook (Mustad 39960D) attached to a floating drum line. Circular hook are designed to hook in the corner of the jaw, facilitating their removal. When removal is not possible, non-stainless hook rusting may facilitate their fall. Captured animals were processed within a cloth harness alongside a small runabout, where total length, sex and maturity were determined. The use of a harness minimize animal stress by covering the eyes and by limiting movements and possibilities of injuries. Maturity state was determined for males based on the extension and calcification of the intromittant organs (claspers); for females it was extrapolated from total length according to Robbins (2006). The V16

transmitter was surgically implanted in the peritoneal cavity. Hooks were removed from sharks before release when possible.

Acoustic array deployment

Sixty-two VR2W acoustic receivers (VEMCO Ltd., Halifax, Canada) were deployed from July 2015 to December 2018 across four regions of the New Caledonian archipelago (D'Entrecasteaux, Chesterfield, Great Northern Lagoon [GNL], Noumea; Fig. 1). Eight additional receivers were deployed along the west coast of New Caledonia to monitor the movements across the study area. Receivers were moored on the reef slope at 20 m depth, approximately 1 m from the substrate facing upward. This configuration has previously been found to be suitable for monitoring grey reef sharks on coral reefs (Field et al. 2010; Heupel et al. 2010). Moorings consisted of a 3 m-long nylon rope, maintained vertical by 20 cm-wide pressure-proof buoy, and attached to the reef matrix by a 2 m-long galvanized steel chain and shackle. Receivers were attached to the rope with cable ties, and were covered with three layers of antifouling paint to prevent benthic organisms from colonizing the sensor and impairing reception performance (Heupel et al. 2008). Data was downloaded and batteries replaced at least once a year.

References

Heupel, M. R., Simpfendorfer, C. A. & Fitzpatrick, R. Large–Scale Movement and Reef Fidelity of Grey Reef Sharks. *PLoS ONE* **5**, e9650 (2010).

Heupel, M. R., Reiss, K. L., Yeiser, B. G. & Simpfendorfer, C. A. Effects of biofouling on performance of moored data logging acoustic receivers. *Limnol. Oceanogr. Methods* **6**, 327–335 (2008).

Field, I. C., Meekan, M. G., Speed, C. W., White, W. & Bradshaw, C. J. A. Quantifying movement patterns for shark conservation at remote coral atolls in the Indian Ocean. *Coral Reefs* **30**, 61–71 (2010).

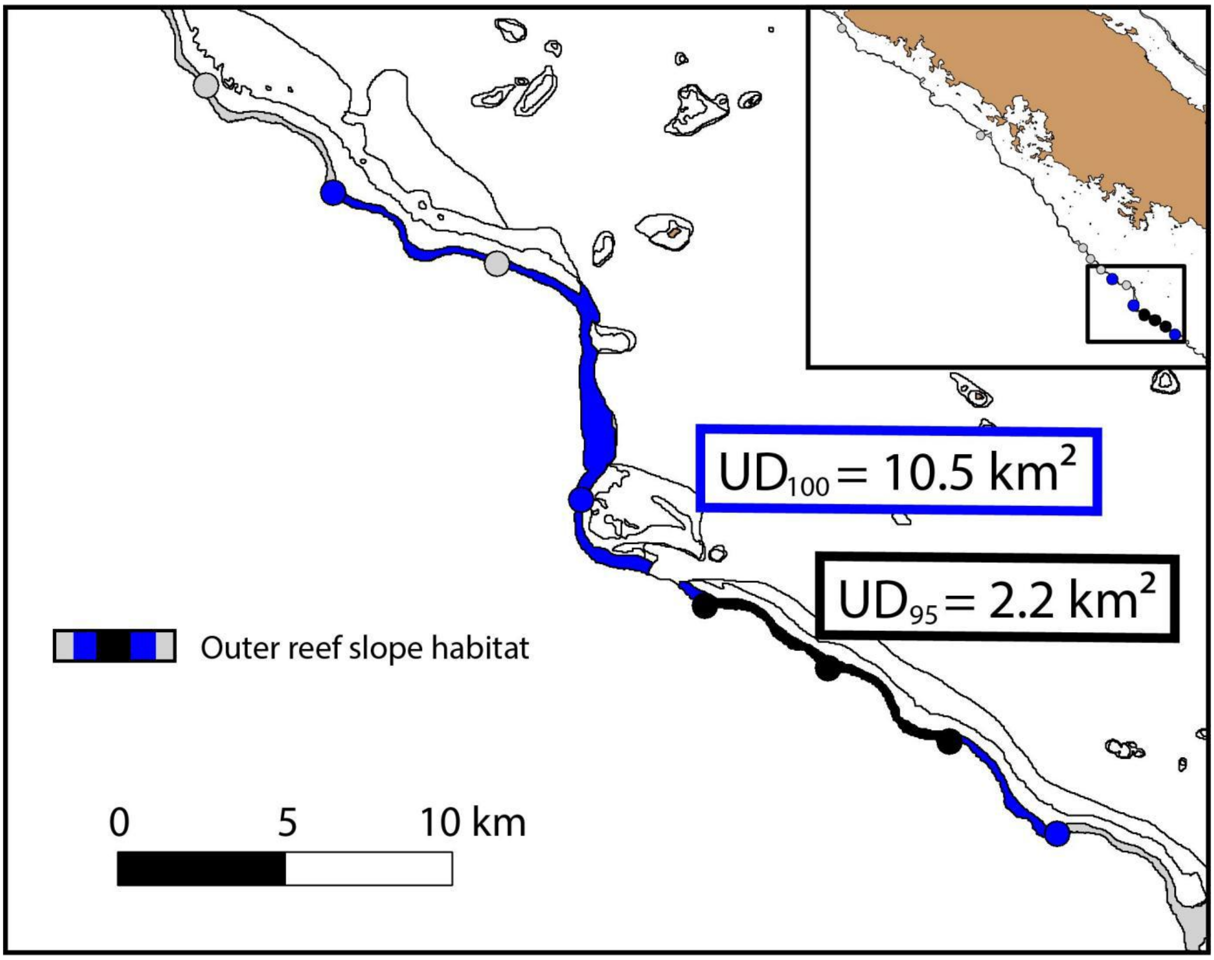

Figure S1. Home range estimation methodology. *The outer reef slope habitat was identified using coral reef habitat data provided by Andréfouët et al. (2014). Acoustic receivers where the individual was detected on 95% of daily occurrences (black dots) and 100% of daily occurrences (black and blue dots) allowed to delimitate* UD_{95} *(black shading) and* UD_{100} *reef portions (black and blue shading). Grey dots represent acoustic receivers not visited by the individual. Map generated with R package rgdal (https://CRAN.R-project.org/package=rgdal).*

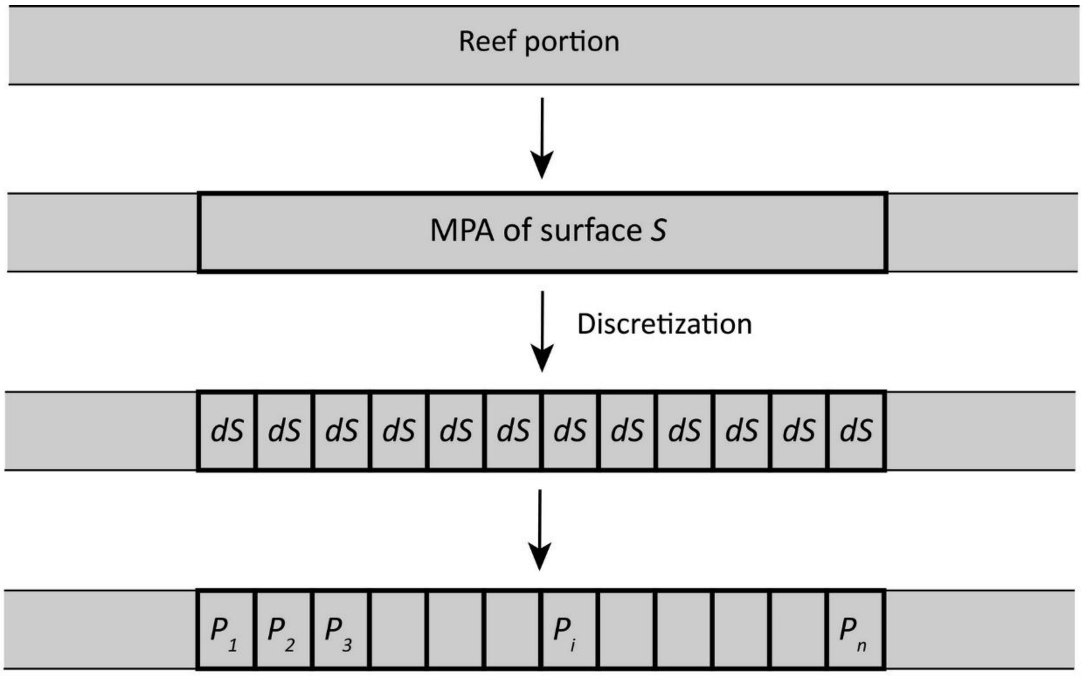

We define Π the probability that the HR of any shark, located at any place in the MPA, would be fully covered

Π is estimated as $$\Pi = \frac{\sum_1^n P_i}{n}$$

with n the number of intervals and P_i the probability that the HR of a shark centred on the interval i would be fully covered by the MPA

P_i is estimated as $$P_i = \frac{\sum_1^N P_{ij}}{N}$$

with N the number of sampled individuals (N = 118 for the entire distribution), and P_{ij} indicating if the HR of the individual j, centred on the interval i, would be fully covered by the MPA

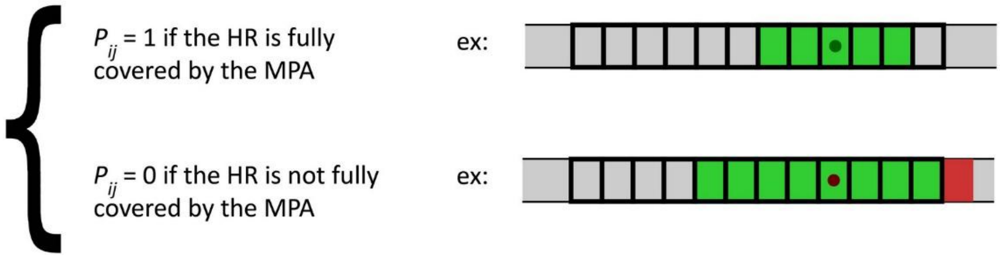

Figure S2. Modelling MPA's ability to cover sharks home range.

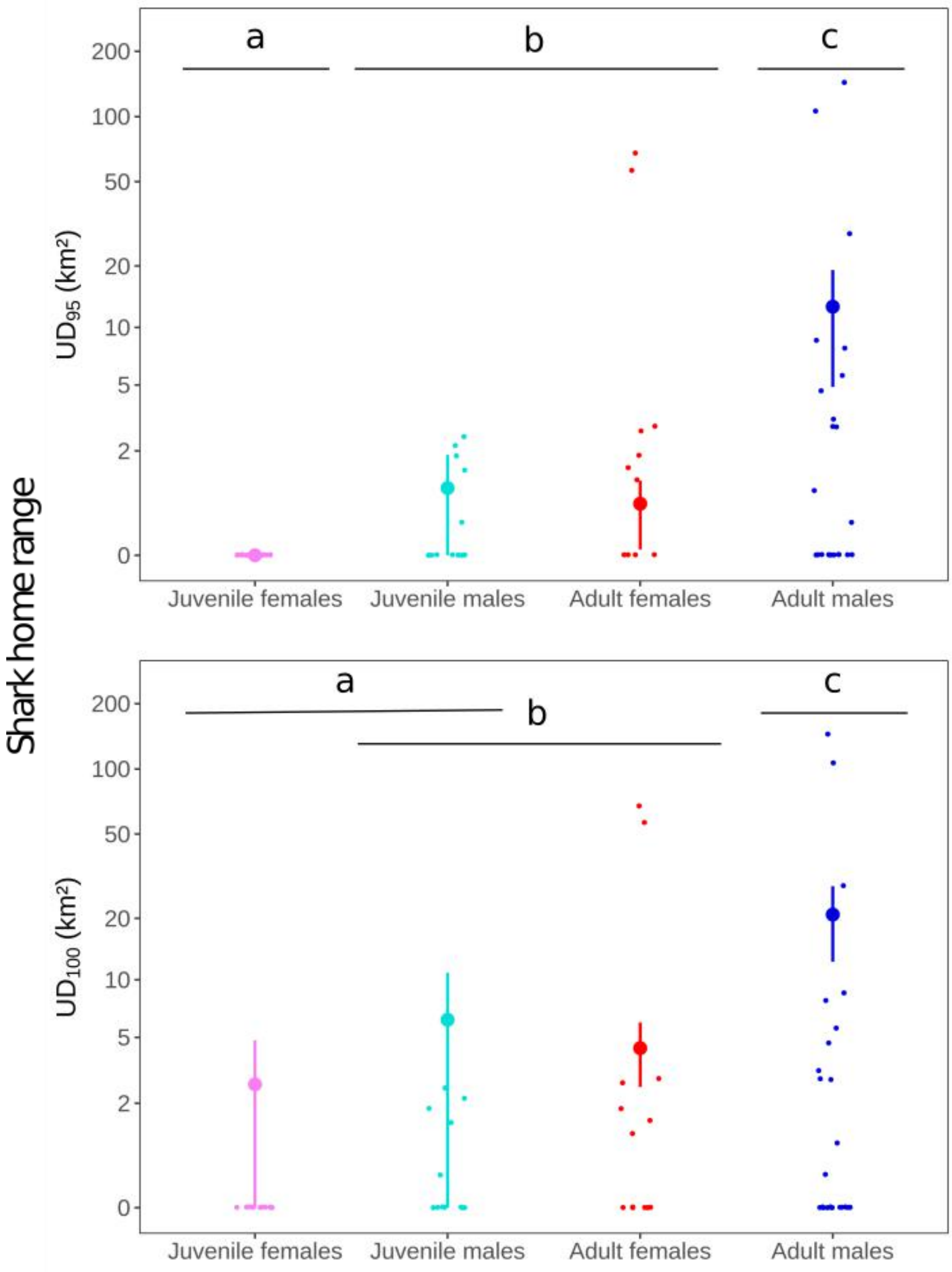

Figure S3. Comparison of home ranges for grey reef shark adults and juveniles of both sexes. *UD_{95} and UD_{100} values represent the surface of outer reef slope habitat encompassed by the 95th and 100th percentile of daily positions. Large dots and bars indicate group means and their bootstrapped 95% confidence intervals. Significance of difference between group means were assessed with pairwise permutation Student tests and displayed with lower case letters. Graphics generated with R package ggplot2 (https://ggplot2.tidyverse.org).*

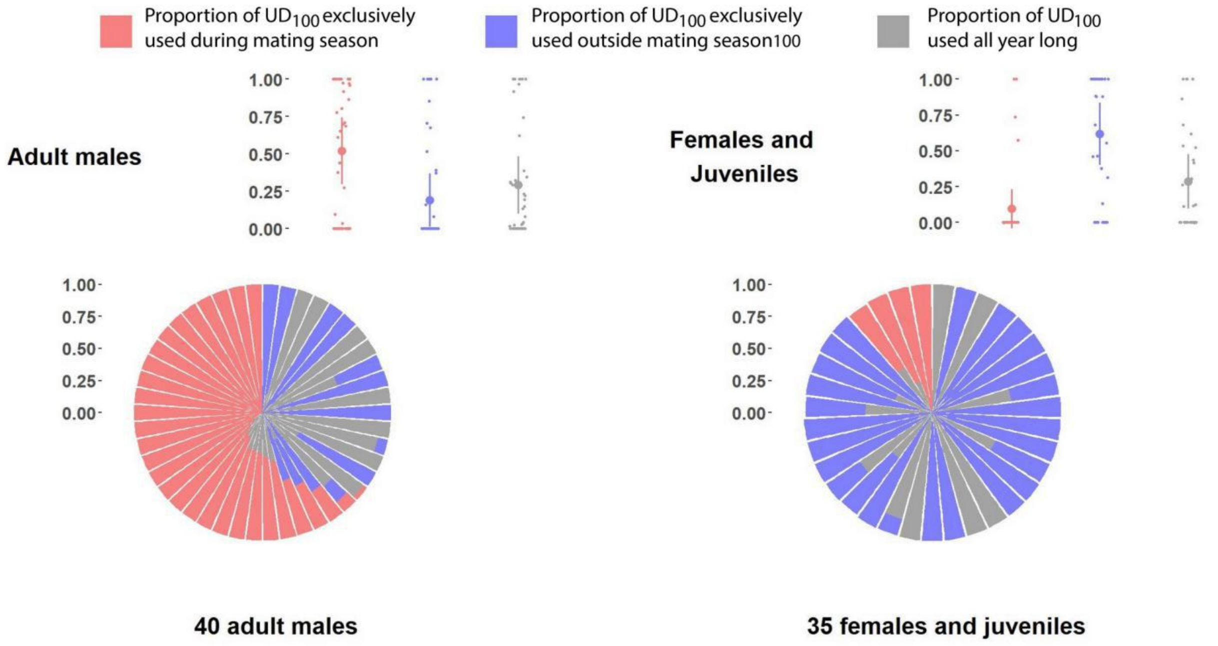

Figure S4. Seasonal space use. *Proportions of outer reef slope portions exclusively used during mating season (July-September), outside mating season and in both season are respectively displayed in red, blue and grey. These proportions are displayed with means and standard deviations for adult males (left) and for females and juveniles (right). Nightingale's roses represent individual proportions. Graphics generated with R package ggplot2 (https://ggplot2.tidyverse.org).*

Home range metric	Season		DF	Sum of squares	Mean square betw.	Iterations	P(perm.)	
UD_{95}	Year-long	Sex	1	5.657	5.657	5000	0.005	**
		Maturity stage	1	4.560	4.560	5000	0.014	*
		Sex : Mat. stage	1	0.650	0.650	224	0.312	
		Residuals	108	91.056	0.843			
	Mating season (July-September)	Sex	1	5.470	5.470	5000	0.010	*
		Maturity stage	1	6.091	6.091	5000	0.014	*
		Sex : Mat. stage	1	3.327	3.327	566	0.150	
		Residuals	108	100.840	0.934			
	October-June	Sex	1	1.433	1.433	1804	0.053	
		Maturity stage	1	0.704	0.704	232	0.302	
		Sex : Mat. stage	1	0.240	0.240	51	0.843	
		Residuals	108	54.481	0.504			
UD_{100}	Year-long	Sex	1	8.218	8.218	5000	0.003	**
		Maturity stage	1	17.577	17.577	5000	0.000	***
		Sex : Mat. stage	1	0.105	0.105	51	0.902	
		Residuals	108	138.335	1.281			
	Mating season (July-September)	Sex	1	11.022	11.022	5000	0.001	**
		Maturity stage	1	7.080	7.080	4875	0.020	*
		Sex : Mat. stage	1	6.054	6.054	3406	0.029	*
		Residuals	108	121.305	1.123			
	October-June	Sex	1	1.695	1.695	639	0.136	
		Maturity stage	1	7.878	7.878	5000	0.015	*
		Sex : Mat. stage	1	4.446	4.446	3444	0.028	*
		Residuals	108	117.506	1.088			

Table S2. Comparison of shark home range by PERMANOVA, excluding migratory individuals. *Six adult males were observed to undergo long-range migrations (Bonnin et al. 2019), up to 300 km from their tagging site. Here, these individuals were removed from the dataset to investigate variations of home range with sex, maturity stage and season. Divergence from the main analysis conclusions (i.e. when including these individuals) is displayed in red.*

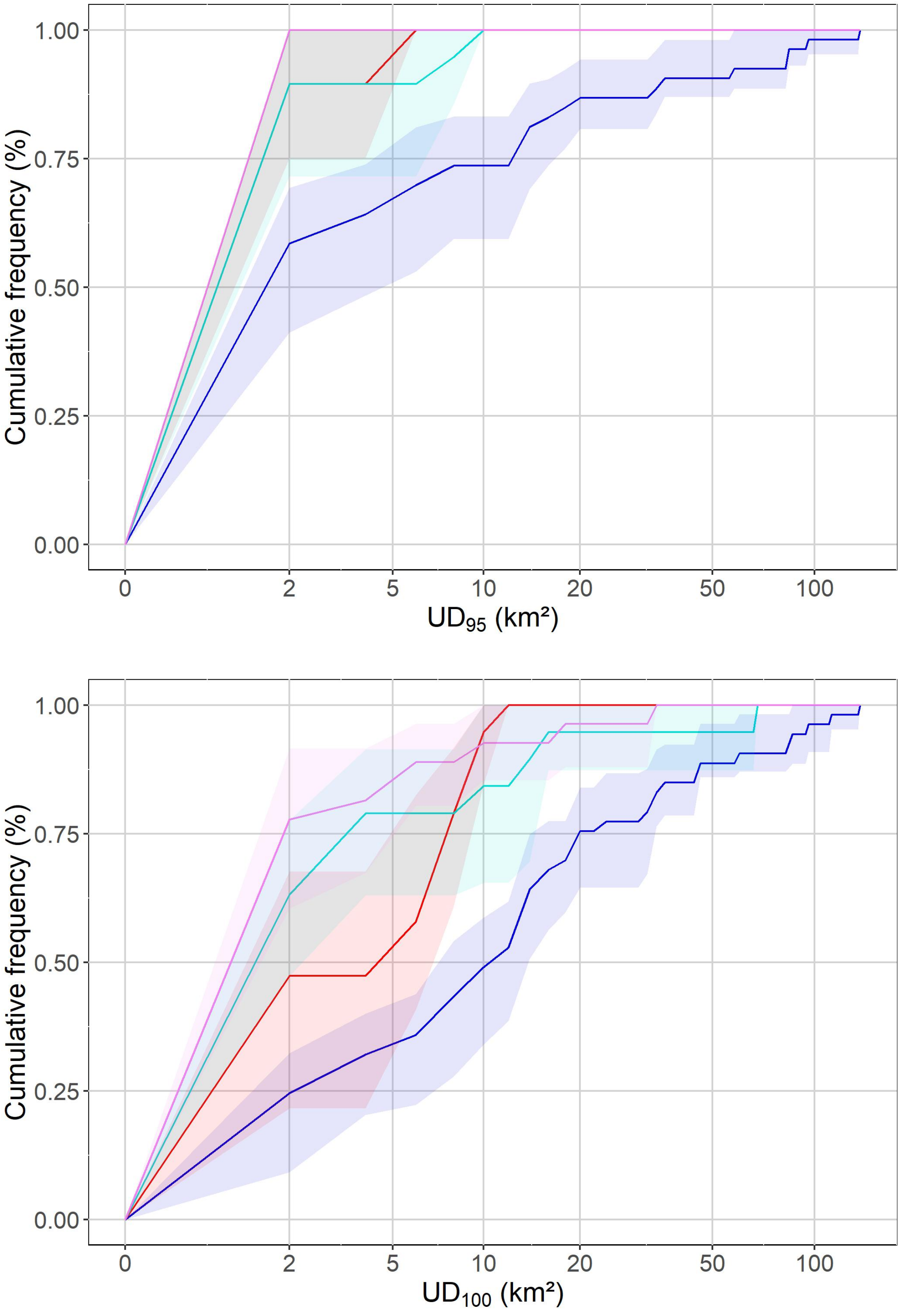

Figure S5. Cumulative distributions of UD_{95} and UD_{100} values. *Cumulative distributions of UD_{95} and UD_{100} values for adult males (dark blue), adult female (red), juvenile males (light blue) and juvenile female (pink) are displayed. Bootstrapped confidence intervals are displayed with corresponding colour shading. Graphics generated with R package ggplot2 (https://ggplot2.tidyverse.org).*

Table S3. Marine Protected Areas' ability to cover grey reef shark home range in New Caledonia. *Values of MPAs' ability to cover sharks home range could not be calculated for Whanga-ledane and Whan-denece reserves, as they were too small to assess considering our home range estimates resolution (i.e. the spacing between acoustic receivers).*

				UD_{95} Protected proportion of…		UD_{100} Protected proportion of…	
**MPA**	**Total Area (km²)**	**Reef area (km²)**	**Covered outer reef slope habitat area (km²)**	**All ind.**	**Adult males**	**All ind.**	**Adult males**
Whanga-ledane	8.7	5.1	1				
Whan-denece Pouarape	2.6	1	1.5				
Ouano	37	11	1.9	74%	56%	44%	23%
Petit Astrolabe	200	5.4	1.9	74%	56%	44%	23%
Poe	32	12	2.1	78%	59%	47%	25%
Tenia	14	6.8	2.8	78%	60%	48%	26%
Dohimen	36	3.5	4.9	80%	63%	51%	29%
Merlet	170	39	5.2	80%	63%	52%	30%
Beautemps-Beaupré	160	18	8	83%	67%	56%	34%
Grand Astrolabe	730	21	8.1	83%	67%	56%	34%
Abore	150	28	9.8	84%	68%	59%	37%
Petrie	600	21	12	84%	69%	62%	40%
D'Entrecasteaux atolls	3500	170	80	94%	87%	87%	78%
*North-Chesterfield	6580	500	280	98%	96%	96%	93%
Chesterfield and Bellona atolls	27150	1470	1130	100%	99%	99%	98%

*The North-Chesterfield no-entry reserve is part of the Chesterfield & Bellona atolls MPA.

S6. Oceanic travels.

Methods

For each pair of reefs encompassed by our array, the shortest possible deep-sea channel(s) connecting them was (were) identified. Coral reef habitat typology provided by Andréfouët et al. (2004) was used to determine oceanic habitat. Nine deep-sea channels were thus identified, spanning from 2 to 422 km. For each of these deep-sea channels, the total number of corresponding crosses were counted, and the corresponding individuals were identified.

Results

Fifty oceanic travels, undertook by 11 individuals, were recorded. All of these travels were recorded between the different D'Entrecasteaux atolls. No movement was recorded between the mainland, Chesterfield and D'Entrecasteaux atolls. Longest recorded oceanic travels were between Portail and Surprise atolls (9.9 km; one travel) and between Huon and Grand Guilbert atolls (9 km; nine travels undertook by five individuals). Individuals that undertook oceanic travels were mostly adult males (n=9), with one juvenile male and one juvenile female.

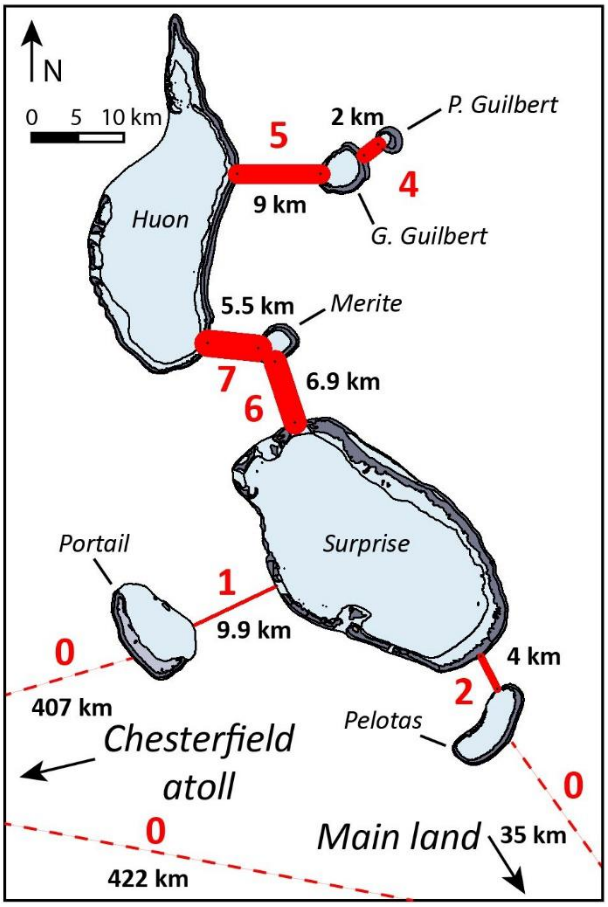

Figure S6. Oceanic travels. *All oceanic channels crossable and recordable with our acoustic receiver array are displayed, with dashed lines if no shark was observed crossing, and with solid lines otherwise. In the latter case, the number of corresponding sharks is displayed. Length of channels are displayed. Huon, Grand Guilbert, Petit Guilbert, Merite, Surprise, Portail and Pelotas atolls form the D'Entrecasteaux atolls group pictured here. Map generated with R package rgdal (https://CRAN.R-project.org/package=rgdal).*
